# Noncontact Sensing of Contagion

**DOI:** 10.3390/jimaging7020028

**Published:** 2021-02-05

**Authors:** Fatema-Tuz-Zohra Khanam, Loris A. Chahl, Jaswant S. Chahl, Ali Al-Naji, Asanka G. Perera, Danyi Wang, Y.H. Lee, Titilayo T. Ogunwa, Samuel Teague, Tran Xuan Bach Nguyen, Timothy D. McIntyre, Simon P. Pegoli, Yiting Tao, John L. McGuire, Jasmine Huynh, Javaan Chahl

**Affiliations:** 1 School of Engineering, University of South Australia, Mawson Lakes Campus, Adelaide, SA 5095, Australia; ali_al_naji@mtu.edu.iq (A.A.-N.); asanka.perera@mymail.unisa.edu.au (A.G.P.); danyi.wang@mymail.unisa.edu.au (D.W.); Leeyh005@mymail.unisa.edu.au (Y.H.L.); titilayo.ogunwa@mymail.unisa.edu.au (T.T.O.); samuel.teague@mymail.unisa.edu.au (S.T.); tran_xuan_bach.nguyen@mymail.unisa.edu.au (T.X.B.N.); timothy.mcintyre@unisa.edu.au (T.D.M.); simon.pegoli@mymail.unisa.edu.au (S.P.P.); yiting.tao@mymail.unisa.edu.au (Y.T.); john.mcguire@mymail.unisa.edu.au (J.L.M.); jasmine.huynh@unisa.edu.au (J.H.); Javaan.Chahl@unisa.edu.au (J.C.); 2 School of Biomedical Sciences and Pharmacy, University of Newcastle, Newcastle, NSW 2308, Australia; loris.chahl@newcastle.edu.au; 3 The Chahl Medical Practice, P.O. Box 2300, Dangar, NSW 2309, Australia; polash_cuet@yahoo.com; 4 Electrical Engineering Technical College, Middle Technical University, Al Doura, Baghdad 10022, Iraq; 5 Joint and Operations Analysis Division, Defence Science and Technology Group, Melbourne, VIC 3207, Australia

**Keywords:** COVID-19, vital signs, remote sensor, thermal camera imaging, video camera imaging

## Abstract

The World Health Organization (WHO) has declared COVID-19 a pandemic. We review and reduce the clinical literature on diagnosis of COVID-19 through symptoms that might be remotely detected as of early May 2020. Vital signs associated with respiratory distress and fever, coughing, and visible infections have been reported. Fever screening by temperature monitoring is currently popular. However, improved noncontact detection is sought. Vital signs including heart rate and respiratory rate are affected by the condition. Cough, fatigue, and visible infections are also reported as common symptoms. There are non-contact methods for measuring vital signs remotely that have been shown to have acceptable accuracy, reliability, and practicality in some settings. Each has its pros and cons and may perform well in some challenges but be inadequate in others. Our review shows that visible spectrum and thermal spectrum cameras offer the best options for truly noncontact sensing of those studied to date, thermal cameras due to their potential to measure all likely symptoms on a single camera, especially temperature, and video cameras due to their availability, cost, adaptability, and compatibility. Substantial supply chain disruptions during the pandemic and the widespread nature of the problem means that cost-effectiveness and availability are important considerations.

## 1. Introduction

COVID-19 cases emerged in late December 2019 as an outbreak of viral pneumonia with cough, fever, and fatigue [1]. It has since spread rapidly throughout the world with hundreds of thousands of reported cases and is now a major global public health threat [2]. It was declared a pandemic on 11 March 2020, by the World Health Organization (WHO) [3]. As of late March 2020, the number of cases worldwide was more than a million and the average death rate was 4.5%, with considerable variation between countries depending on the availability of medical services and public health measures [4].

The causative agent of COVID-19 was identified as coronavirus 2 (2019-nCoV) [5], later designated SARS-CoV-2 [6]. Coronaviruses are non-segmented, positive-stranded ribonucleic acid (RNA) viruses surrounded by a protein envelope with characteristic crown-like spikes on their surfaces. The coronaviruses that infect humans by cross-species transmission pose a major threat to human health and have caused two serious outbreaks of acute respiratory syndrome in the past two decades, severe acute respiratory syndrome (SARS) in 2003, and Middle East respiratory syndrome (MERS) in 2012.

## 2. Epidemiology

An understanding of the epidemiology of COVID-19 is evolving as the pandemic progresses and more information should become available in the coming months. Although the early cases had some contact with the seafood (wet) market, it soon became clear that human-to-human transmission occurred [7]. COVID-19 has been found to have higher levels of transmissibility and pandemic risk than SARS [8]. Guidelines from Chinese health authorities in early February 2020 stated an average incubation duration of seven days, ranging from two to 14 days [7].

It also became clear early in the epidemic that the elderly were more susceptible, whereas cases in persons under 18 years of age were few. Pediatric patients have been found to have only mild to moderate symptoms of COVID-19, and many children were asymptomatic [9]. A further study confirmed these findings and proposed that children could be facilitators of transmission of COVID-19 [10]. The authors further suggested that there is an urgent need to investigate the role of children in the transmission of COVID-19.

## 3. Clinical Features of COVID-19

The most common early symptoms and signs of COVID-19 include fever, dry cough, sore throat, and fatigue. Less common symptoms are diarrhea, muscle aches, and headache, indicating that multiple systems are involved. In a proportion of patients, reportedly about 39% [1], the disease progresses to shortness of breath and acute respiratory distress syndrome (ARDS), requiring mechanical ventilation. Multiple organ failure causing death occurs in a small proportion of patients.

A study of 191 patients from two hospitals [11] showed increasing probability of death was associated with older age, higher Sequential Organ Failure Assessment (SOFA) scores (used to determine the rate of organ failure), and levels of d-dimer (fibrin degradation product, indicative of thrombosis) greater than 1 µg/mL. Higher levels of comorbidities such as hypertension, diabetes, coronary heart disease, and chronic obstructive lung disease were found in non-surviving subjects [11]. Development of heart failure has been identified as a particular risk in those patients with underlying cardiovascular disease [12]. Lower mortality has been reported by early detection and intervention for those patients at risk of serious illness [13].

The particular vulnerability of the cardiovascular system may be related to the ability of the spike protein of the coronaviruses to bind to angiotensin converting enzyme 2 (ACE2), a transmembrane aminopeptidase that is highly expressed in the heart and lungs and plays a vital role in the cardiovascular and immune systems [14]. The finding that ACE2 is a functional receptor for coronaviruses and acts as an entry point for the virus into cells and the fact that ACE2 is elevated in patients with cardiovascular disease [15] may explain the greater vulnerability of these patients.

As yet, there is no known effective treatment for COVID-19 [16]. The current treatment of patients is symptomatic, aimed mainly at supporting the cardiopulmonary system. The effect of a combination of the antiviral drugs, lopinavir and ritonavir, which was found to result in less adverse clinical outcomes during the SARS epidemic, is currently under trial [17]. However, current research is primarily directed at finding a vaccine to prevent infection with COVID-19.

The long-term effects of COVID-19 remain unknown. However, in a 12 year follow-up study, subjects who recovered from SARS were found to have chronic cardiovascular damage and disorders of lipid and glucose metabolism [18]. The similar structure of the coronaviruses causing SARS and COVID-19 raises the possibility of chronic cardiovascular damage in patients with COVID-19 and the importance of cardiovascular protection during treatment of patients [18].

## 4. Diagnosis of COVID-19

The first definitive test for COVID-19 was Reverse Transcriptase Polymerase Chain Reaction (RT-PCR), which detects SARS-CoV-2 nucleic acid (RNA). This test was developed very early after identification of the causative virus. However, RT-PCR takes time and requires specialized laboratories and trained staff. Recently, serology antibody tests have been developed that detect IgG and/or IgM antibodies to SARS-CoV-2. These tests use finger-prick blood samples and provide results in 15–30 min. Their limitation is that it takes several days after an individual becomes infected for the antibodies to become detectable. Since COVID-19 is an emerging viral infectious disease, there is limited evidence currently available to assess the accuracy of these new tests [19].

Comparison of serial RT-PCR assays and chest computer tomography (CT) scan results obtained from patients with suspected COVID-19 showed that chest CT scans had a higher sensitivity than RT-PCR for diagnosis of COVID-19 [20]. Therefore, in the hospital setting, CT scans would be most useful in early diagnosis of suspected cases.

## 5. Symptoms of COVID-19 Detectable by Noncontact Sensors

The highly contagious nature of COVID-19 and the ongoing problem of detecting the signs of the infection in large groups of people such as travelers makes the rapid detection of the signs by noncontact sensors an important goal. The early clinical signs of COVID-19 are fever, cough, and fatigue. Later signs that have been reported in hospitalized patients are increased heart rate, respiratory rate, and ocular signs. Those infected with COVID-19 who are seriously ill with inadequate oxygenation may have cyanosis, which is exhibited as a blue color of the body and visible mucosa [21]. Fever, cough, fatigue, ocular signs, and increased heart rate and respiratory rate are discussed further below.

### 5.1. Fever

Normal body temperature varies between individuals and with age, activity, and time of day. The average normal body temperature is generally accepted as 37 °C, although it has a wide range from 36.1 to 37.2 °C. Fever is commonly accepted as a body temperature over 38 °C, indicating infection or illness [22]. A recent study of hospitalized patients with COVID-19 showed that 98% of patients had temperatures above 37.3 °C and 78% had temperatures above 38 °C [17].

The presence of elevated body temperature (fever) is the early sign that is currently widely used as a screening method for detecting COVID-19 infection in groups of people such as travelers. Early advice from the WHO was that body temperature screening detected the majority of cases of COVID-19 [23].

Many different thermometry methods are used to detect fever in clinically ill subjects, including oral, rectal, axillary, auditory meatus, and tympanic membrane measurements. The method commonly used for non-invasive, rapid screening of large numbers of people is forehead infrared body temperature measurement. A study conducted using data obtained during the SARS epidemic in Taiwan, comparing infrared body thermometry with measurements obtained with a tympanic thermometer, showed that measurements of temperature from the auditory meatus but not the forehead correlated with tympanic temperature measurements [8]. A much higher rate of false-negative results was also reported for forehead measurements compared with auditory meatus measurements [8]. Despite the limitations of forehead infrared thermometry, it remains the most widely used screening method.

The adequacy of body temperature measurement as a screening method for detection of COVID-19 has been questioned, not only on methodological grounds, but also as a result of epidemiological studies in which cases of asymptomatic subjects transmitting the disease have been suspected [24]. The possibility of asymptomatic and presymptomatic transmission has recently been studied [25]. From the data available to date, the authors concluded that, based on serial intervals defined as the time duration between a primary case developing symptoms and a secondary case developing symptoms, it was likely that presymptomatic transmission occurred in about 10% of cases of COVID-19 [25]. The role of the asymptomatic transmission is less clear, but there is some evidence that this might also occur [25,26].

Thus, there are two major limitations to the use of fever for the detection of COVID-19, the limitations regarding the detection of elevated body temperature, and the possibility of asymptomatic subjects. As was suggested following the SARS epidemic [8], screening for other signs such as cough and cardiopulmonary changes would be necessary to reduce the risk of non-detection of COVID-19 cases.

### 5.2. Cough

A study of forty-one hospitalized patients with COVID-19 showed that 76% had cough [17]. Subjects with cough are highly likely to spread infection, and therefore it is important to screen for cough in groups of people. Coughing involves a characteristic explosive sound and movement of the rib cage that might be sensed remotely.

### 5.3. Fatigue

Fatigue is a subjective sensation that might not be detectable by sensors until subjects are more severely ill and exhibit objective signs. In these subjects, multiple facial morphological features such as those reported by Li et al. [27] might be used to detect fatigue.

### 5.4. Ocular Signs

Ocular signs of irritated eyes including conjunctival hyperemia, swollen eyes, and increased tear production have been reported in about 30% of patients with diagnosed COVID-19 [28]. Evidence of irritated eyes, such as rubbing of the eyes, might be detected by sensors in a fraction of subjects early in the disease.

### 5.5. Respiratory Rate and Heart Rate

The normal respiratory rate for adults at rest ranges from 14 to 20 breaths per minute. It has been reported that respiratory rate is a commonly neglected vital sign, which may be a predictor of clinical outcome [29]. Recent evidence suggests that a subject with a respiratory rate greater than 20 breaths per minute is probably unwell, and a subject with a respiratory rate greater than 24 breaths per minute is likely to be critically ill [29].

Dyspnea is a subjective sensation, also known as shortness of breath. A common symptom of COVID-19 is dyspnea, which may manifest as an increase in respiratory rate and heart rate. In an early study on hospitalized patients with COVID-19, 55% had dyspnea, and about half of those had a respiratory rate greater than 24 breaths per minute [17]. In a further study of 44,672 diagnosed cases of COVID-19, 14% had dyspnea and a respiratory rate greater than 30 breaths per minute [30].

The mean normal heart rate for adults is 72 beats per min. In a study on the effects of acute febrile infection on cardiac rhythm in young adult males, it was shown that a body temperature rise of 1 °C caused a heart rate increase on an average of 8.5 beats per minute [31].

Respiratory rate and heart rate are vital signs that would prove useful in screening for subjects with suspected COVID-19.

## 6. Technology Used for Measuring Vital Signs

Human vital signs such as temperature, breathing rate (BR), heart rate (HR), heart rate variability (HRV), blood oxygen saturation (SpO_2_), and blood pressure (BP) indicate human state of health to a large extent [32,33]. COVID-19 effects on vital signs, as shown above, are significant on cardiorespiratory state and temperature. Certain other characteristics such as coughing and conjunctivitis are clearly detectable by video camera under the right circumstances, while being difficult to detect remotely using non-imaging technology. Coughing can be detected acoustically. This review focuses on symptoms that might be detected using noncontact sensing technology which are the internal parameters of temperature and cardiorespiratory rates.

There are various non-contact methods for measuring vital signs remotely, including by magnetic induction, the Doppler effect with radar or sonar, video camera imaging, and thermal imaging [34]. These techniques have been shown to be effective in remotely monitoring vital signs with acceptable accuracy, reliability, and sometimes practicality [34]. Each method mentioned above has its pros and cons and may outperform in some challenges but be inferior in others. Doppler radar is highly affected by motion artefacts, can have biological effects on humans, and is not suitable for monitoring multiple subjects simultaneously. Visible spectrum and thermal spectrum cameras offer the best options of those studied, thermal cameras due to their potential to measure all likely symptoms on a single camera, especially temperature, and video cameras due to their availability, cost, adaptability, and compatibility.

### 6.1. Thermal Imaging Technology

Infrared thermography (IRT), also known as thermal imaging, is a promising monitoring and diagnostic technique in the medical field [35]. For example, thermal imaging can be used to assess HR and BR, endocrinological disorders (especially diabetes mellitus), vascular disorders, neurological disorders, musculoskeletal disorders, oncology, regenerative medicine, and surgery. Additionally, a popular application of thermal imaging is screening persons with fever at airports, schools, hospitals, etc. Therefore, IRT is a passive, non-contact monitoring technique, which senses the radiation naturally emitted from an object such as human skin. Thermal imaging does not require an illuminating radiation source or a dedicated light source. These are the most significant advantages of thermal imaging over other non-contact techniques. They are not affected by illumination variation, can work in darkness, and are difficult to falsify using makeup or masks [36].

**Figure 1:**
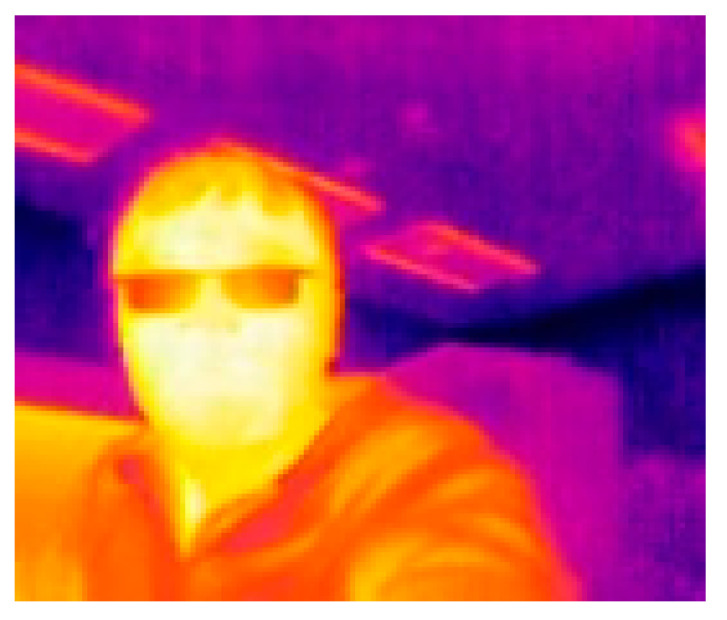
Thermal image captured by thermal camera.

Thermal cameras respond to wavelengths starting at around 3 micrometers (µm) compared to visible wavelengths, ending at about 0.75 µm. An array of thermally sensitive elements behind a specified lens produces an image much like an optical camera, with higher radiation measured from hotter objects. This leads to a grey image, with lighter areas being warmer rather than darker. Depending on the application, this scale might be reversed for human operators. The use of “false color” is common in thermal images and thermal video, because it allows for increased ability for human operators to discern temperature. Colder temperatures are often assigned a shade of purple, blue, or green, whereas hotter temperatures can be given a shade of orange, red, or yellow [37]. Figure 1 shows a thermal image captured by a thermal camera where the person is covered in shades of orange and yellow, whereas other areas are blue and purple. That is because he is radiating more heat than surrounding objects. False color is purely a remapping of grey levels and is not a feature of the underlying thermal images.

Thermal cameras work on the principle that all objects that have a temperature above absolute zero (−273.15 °C) emit electromagnetic energy, also known as infrared radiation or thermal radiation [38].

To accurately observe the temperature of a target using a thermal camera, the target should be an ideal black body. A black body in thermal equilibrium will absorb all incident electromagnetic radiation and emit an equal amount of energy through isotropic radiation. The distribution in wavelength of the emitted radiation is governed by the temperature of the body; thus, as the temperature increases, the distribution shifts towards shorter wavelengths. This phenomenon is described by Planck’s law. A thermal camera measures the predominant wavelength of the radiation emitted by a body. The human body is not an ideal Plank black body radiator but is a close approximation in some bands [38]. It is generally accepted that the emissivity of human skin in the IR range of 3–14 µm is 0.98 ± 0.01 [38], which, while close to that of a black body, can cause disturbances due to non-ideal radiation emission and reflections. Thus, there will always be some degree of ambiguity when measuring skin temperature through thermal imaging. In a controlled environment, this can be accounted for with calibration. In uncontrolled environments, however, it is significantly more difficult to calibrate.

This is just one of many limitations that arise when using thermal cameras for the detection of elevated body temperature. Infrared camera manufacturers, such as FLIR [39], state that such technology can only be used to detect skin temperature (as opposed to core body temperature) and must be operated in a controlled environment to do so. The temperature must be in range of 20 to 24 °C, and the relative humidity should be in the range from 10% and 50%. The temperature measured on the skin’s surface is offset from the subject’s core body temperature; hence, measurements must be calibrated to be indicative of body temperature. Environmental, operational, and subjective factors such as convective airflow, reflective surfaces, IR contamination (from sources such as sunlight and heaters for example), the sweat produced by the subject, wearable accessories covering some part of the face (such as glasses or baseball caps), ambient temperature, humidity, and emissivity all have an effect on the acquired data. Thus, thermal imaging may be used to determine anomalies relative to other subjects; however, there are many factors that prevent determination of the absolute core body temperature of a given subject from range. It is suggested that the area medially adjacent to the inner canthus of the eye provides the most consistent measurement, as determined with mass screening [40].

#### 6.1.1. Body Temperature Measured with Thermal Camera

**Figure 2:**
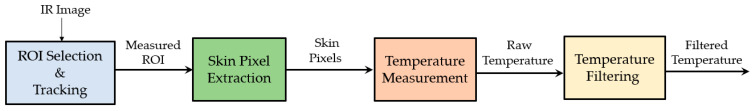
The non-contact body temperature measurement framework proposed in [41].

In the literature, thermal cameras have been used by numerous researchers to extract body temperature, as shown in Table 1. For example, Bilodeau et al. [41] proposed a non-contact method to measure the body temperature of a moving animal or human in a laboratory setting based on IRT using a thermal camera. As shown in Figure 2, a region of interest (ROI) was selected manually and tracked automatically with a particle filter. A Raw temperature signal was measured from extracted skin pixels. To minimize camera measurement noise, a Kalman filter was applied to the raw signal. However, in the proposed method, the ROI was selected manually.

Aubakir et al. [42] used a long wavelength infrared (LWIR) camera to monitor body temperature based on an automatic face detection using the Viola–Jones (V–J) algorithm and ROI averaging. It is noted that this method of ROI averaging is prone to perturbances due to environmental factors. Another study by Sharma et al. [43] proposed a contactless approach to measure temperature using the Viola–Jones face detection algorithm. Authors used an algorithm to select the best frame containing all the features of the face rather than computing the temperature of the face in each frame and then calculated the temperature for the same frame. However, the Viola–Jones face detection method was sensitive to the subject’s head movement.

**Figure 3:**
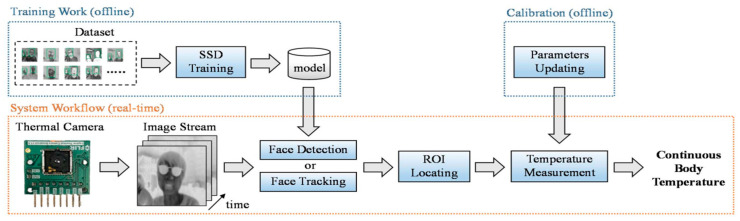
The thermal camera-based continuous body temperature measurement framework proposed in [44].

Lin et al. [44] presented a non-contact, automatic continuous body temperature measurement (CBTM) system using a thermal camera. In the proposed method depicted in Figure 3, face detection was implemented using a neural network, and a kernel correlation filter (KCF) tracker was used to track the face region across each frame. Nevertheless, in the proposed method, participants were advised to sit and remain stationary, making the system not applicable for real-time situations.

Sumriddetchkajorn et al. [45] introduced a parallel measurement technique to screen human temperatures that is applicable to large public areas by combining infrared technology, image processing techniques, and human flow management. Through image filtering, morphological operations, and particle filtering, large numbers of subjects were able to be processed efficiently. With 100% sensitivity and 36.4% specificity, however, this approach requires refinement.

**Figure 4:**
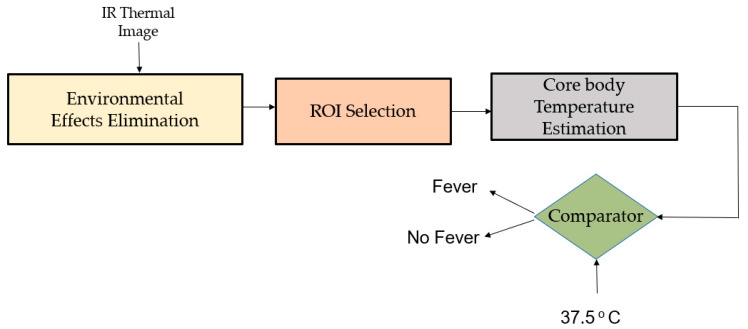
Block diagram of fever screening process proposed in [46].

Using an infrared thermal camera, Silawan et al. [46] proposed a novel idea to estimate core body temperature. The method enhances the sensitivity and specificity of a fever screening system considering various environmental situations (Figure 4).

A pilot study by Thomas et al. [47] explored the use of an IR camera for measuring the core body temperature of workers in hot work environments. The strong correlation between the measured and reference data showed potential for the infrared camera as a non-invasive alternative to the thermometer pill and rectal thermometer.

#### 6.1.2. HR and BR Measured with Thermal Camera

To assess a human’s health condition, especially to identify a virus-infected patient, measuring body temperature alone is not sufficient. Other vital signs, such as breathing rate and heart rate, should be considered as well. Thermal imaging techniques can detect two physiological phenomena caused by cardiorespiratory activity. The first phenomenon related to cardiac activity is the slight heat differences produced by the pulsating blood flow in the major superficial arteries in particular anatomical regions. A thermal camera can detect these heat differences to extract the pulse signal. The heat difference due to the pulsating blood flow is approximately 0.08 K, which is far less than the temperature of normal skin (310 K) from a black-body perspective [48]. The second phenomenon related to breathing activity is the small temperature differences around the nostrils of approximately 0.1 K [49] due to the heat transfer from warm exhaled and cold inhaled lung contents.

A summary of studies in the literature where thermal cameras were used by researchers to monitor the breathing rate and heart rate is shown in Table 2. Murthy et al. [50] proposed a noncontact technique to monitor breathing rate using thermal imaging based on a statistical methodology that modelled breathing as a combination of expiration and non-expiration distributions. Fei et al. [51,52] enhanced the thermal imaging technique by using an optical bandpass filter and Fourier analysis based on the fast Fourier transform (FFT). In these studies, the ROI selection was done manually, and there was not sufficient compensation for motion.

**Figure 5:**
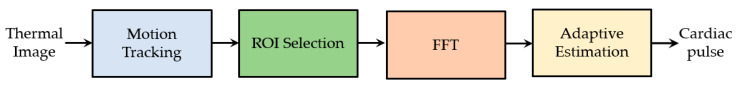
Block diagram of the cardiac pulse measurement proposed in [53].

Sun et al. [53] presented a novel non-contact technique for measuring a cardiovascular pulse using thermal imaging. The algorithm includes motion tracking, manual ROI selection, FFT, and adaptive estimation to measure cardiac pulse (Figure 5). However, in this study, the subject sample size was very small. Garbey et al. [54] further elaborated the Sun et al. method by increasing the sample size from five to 32 subjects and recording time from two minutes to five minutes. However, both methods are highly affected by the movement of subjects and the manual selection of ROI.

Chekmenev et al. [55] introduced a novel non-contact method to remotely measure HR and BR based on wavelet analysis using continuous wavelet transform (CWT), which gave a better performance than FFT. However, they only considered four subjects to validate their method, and the ROI selection was manual. In another study, Fei et al. [56] introduced a new and enhanced technique to monitor BR based on automatic tracking/localization of ROI using a coalitional tracking algorithm and wavelet analysis based on CWT. Using CWT, not only the breathing rate, but also the full breathing waveform was recovered.

Shakhih et al. [57] presented an approach to measure the time of the inspiration (TI) and expiration (TE) considering three different breathing patterns that were simultaneously captured by infrared thermal imaging (ITI) and respiratory inductive plethysmography (RIP). However, the method was highly susceptible to head movement and image processing was also not sufficient.

**Figure 6:**
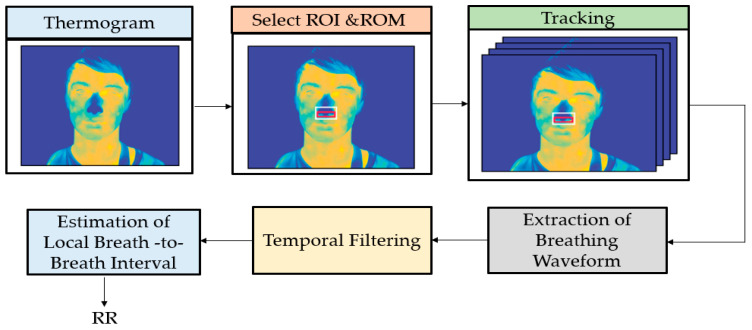
Block diagram for the BR estimation proposed in [58,59].

Pereira et al. [58] presented a new robust method to remotely measure BR by using thermal imaging captured by an LWIR camera. As shown in Figure 6, in the proposed method, the ROI and region of measurement (ROM) were identified automatically, and then tracked using a particle filter framework. A Butterworth bandpass filter was applied to the raw signal. Finally, three estimators were calculated to compute instantaneous breathing frequencies. However, the authors only considered BR.

**Figure 7:**
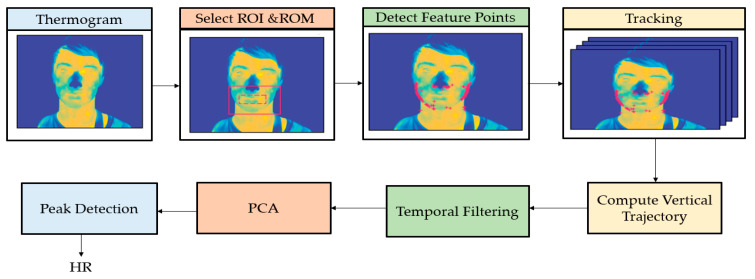
Block diagram of HR estimation proposed in [59].

The authors [59] proposed a non-contact method to monitor cardiorespiratory signals using thermal imaging. BR was extracted incorporating the same algorithm as [58]. As shown in Figure 7, feature points were detected and tracked within the manually selected ROI. Temporal filtering, principal component analysis (PCA), and peak detection were used to estimate HR.

Most of the above-discussed methods used thermal information around the nostril area, which is not suitable for long term monitoring, because it can happen that the nose is not in the field of view of the thermal camera. To deal with this issue, Pereira et al. [60] presented an enhanced and robust method to estimate BR using thermal imaging, considering both the temperature difference around the nostril area and mouth and the movement of the shoulders. In the proposed algorithm, four ROIs were detected automatically and tracked using a particle filter framework. Only five subjects were considered in this study.

**Figure 8:**
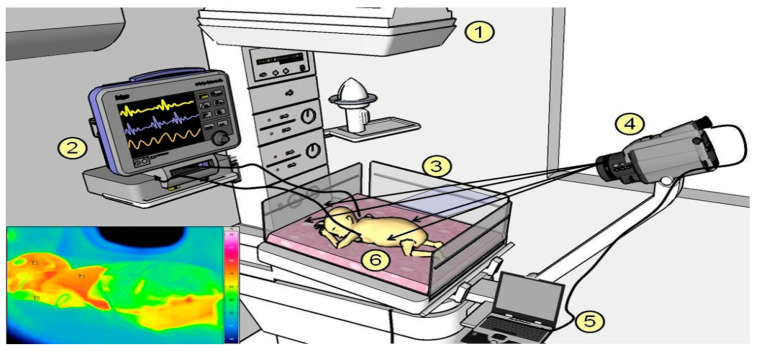
Schematic of experimental set up used in [61] to monitor BR of neonates using IR thermal camera. (1) Radiant warmer bed, (2) bedside monitor, (3) camera field of view (FOV), (4) IR thermal camera, (5) analysis workstation, and (6) infant under NIRT imaging.

Most of the works discussed above considered only adults as their subjects to monitor vital signs. However, a few works did research on infants as well. For example, Abbas et al. [61] first used infrared thermography to monitor the breathing rate of neonates in the neonatal intensive care unit (NICU) using an infrared camera set up shown in Figure 8 and an algorithm based on CWT. However, they did not consider a tracking algorithm, and the sample size was small. Pereira et al. [62] proposed a feasible approach based on IRT to monitor the BR of preterm babies incorporating the same algorithm as [58]. However, the sample size was very small, as they considered only four infants. In [63], the authors presented a “black-box” or grid-based approach algorithm to remotely monitor the BR of neonates in the NICU using an LWIR camera. However, this method is highly affected by motion artefacts.

#### 6.1.3. Image Processing Techniques Associated to Thermal Imaging

Thermal imaging systems use several image processing techniques to extract the required image details from the thermal images. These can range from low level image processing to high-level deep learning-based techniques. We gather the widely used methods for image analysis in thermal imaging.

Having a reliable object detection model is very important for the prediction of objects on thermal images. For detection in thermal infrared, hotspot detection or thresholding has historically been the major approach [64]. Thresholding combined with post-processing (e.g., merging and splitting of blobs) is an efficient detection technique in the case of high background/object contrast, a situation more or less common depending on the application [65,66].

In most cases in the literature for thermal infrared images, ROIs are characterized manually because of the absence of reliable face and facial landmark detection algorithms. In other cases, ROIs are defined semi-automatically or automatically; however, this is done with the condition that the full-frontal view or a certain image area of the face is given [67]. Some detection methods exploit the advantages of the visual and thermal modalities, respectively, by combining information extracted from visual and thermal imagery of the same scene [68,69,70].

Recent advances in deep convolutional neural networks have helped enable sophisticated facial or human body detection and recognition systems, which prove valuable in surveillance and security systems applications [69]. Existing state-of-the-art facial recognition systems have demonstrated high-performance accuracy for automatic object detection and identification/recognition tasks [71,72,73,74,75].

Although there has been great success in object detection and recognition systems in the visible light domain [71,73,76], in other domains like the thermal domain, considerably less work has been done to accomplish such performance. Few notable methods used deep learning methods originally developed for RBG images [75], developing new feature detectors for thermal images [77], thermal imaging datasets with multiple labelled environment conditions [78], and thermal imaging-based augmented vision [79].

Object detection methods have progressed significantly over the years from simple contour-based methods using support vector machines (SVM) [77,80] to those using deep classification models [76,81] that utilize hierarchal representations of data [79].

Due to advances in deep learning, a more generalized form of object detection has evolved over time. Convolutional classifiers have replaced the exhaustive search for classification [79]. Object detection models have been proposed to work with relatively good accuracy on the visible spectrum using models that consist of (i) a two-stage system made up of a classifier connected with a region proposal network (RCNN [82]) and (ii) a single stage network, which performs the classification and localization layers in a cohesive space (YOLO [76] and SSD [81]).

Image segmentation partitions images into multiple parts or regions, often based on the characteristics of the pixels in the image. It could involve separating foreground from background, or clustering regions of pixels based on similarities in color or shape.

Because individuals are usually warmer, they appear brighter than the background in thermal images. Thus, a common point to start to extract human candidates is a single threshold. Calculating the threshold is as simple as finding a difference between the maximum and minimum image intensities and taking the average value of that difference [83]. Deep neural networks have been proposed to perform the semantic segmentation in complex scenes recorded by synchronous RGB and thermal cameras [84,85]. However, these networks require a large number of annotated data to train them.

The main objective of image translation is to learn one or multiple mappings between the source domain and the target domain. This can be realized in both supervised [86] and unsupervised [87] ways, or even in semi-supervised ways [88].

**Figure 9:**
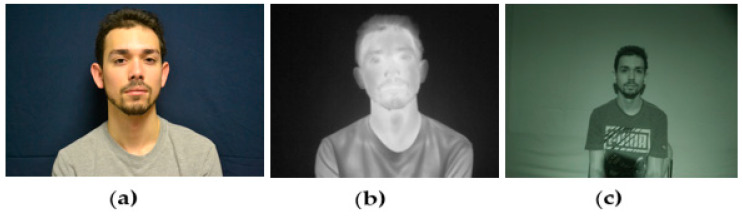
Sample cross-modality images of three domains from the Tufts Face Database [89]. (**a**) Visible light, (**b**) thermal, and (**c**) near infrared (NIR).

Face recognition studies in the visible light domain (VLD) have achieved impressive performances due to the availability of massive datasets. With these great strides, it is imperative to extend the existing VLD based face recognition systems into other less studied domains such as near-infrared imaging (low-light) and thermal imaging (no-light) [72]. The different domains are shown in Figure 9. The VLD domain could not be easily replicated in the thermal domain due to the relatively small amount of training data available and the domain gap between thermal and visible light [72].

In recent times, the use of generative adversarial networks (GAN) [90] in arbitrary image-to-image translation applications has shown encouraging results [91]. Using an antagonistic game approach, GANs significantly increased the quality of image-to-image translation. Pix2pix GAN for geometrically aligned image pair translations was presented by Isola et al. [86]. This work indicated phenomenal performance in arbitrary image-to-image translations. The pix2pix was trained by Zhang et al. [92] to transform a human face in a thermal image to a color image. In a cross-modality setting of thermal to visible range, the quality of a face recognition performance was improved by the translation. While the human face has a generally steady temperature, translating an image from color to thermal image for the entire human body with an arbitrary background is more ambiguous and is therefore constrained by the sequence of happenings for an individual [91]. Another prominent development in this area, TV-GAN [72] translates face thermal images into visible light images using a facial identify loss function, and this work can generate naturally lit face images whilst preserving identity.

With the right thermal imaging camera, the right lens, and following the correct guidelines and standards, thermal imaging systems can be effective as a screening tool. However, this is still a challenging and active research area with a lot of ongoing developments. A thorough explanation on the limitations of thermal imaging can be found in [93]. Here, we summarize a few of them:

- Although these systems may be in use for initial temperature assessment to triage individuals in high throughput areas (for example, airports, businesses, and sporting events), the systems have not been shown to be effective when used to take the temperature of multiple people at the same time. It is difficult to find stable solutions related to “mass fever screening”.
- These systems measure surface skin temperature, which is usually lower than a temperature measured orally. Thermal imaging systems must be adjusted properly to correct for this difference in measurements.
- These systems work effectively only when all the following are true:
  o The systems are used in the right environment or location.
  o The systems are set up and operated correctly.
  o The person being assessed is prepared according to instructions.
  o The person handling the thermal imaging system is properly trained.
- Room temperature should be 68–76 °F (20–24 °C) and relative humidity 10–50 percent.
- There are items that could impact the temperature measurement:
  o Reflective backgrounds (glass, mirrors, and metallic surfaces) could reflect infrared radiation.
  o Movement of air in the room, direct sunlight and radiant heat (portable heaters, electrical sources).
  o Strong lighting (incandescent, halogen, and quartz tungsten halogen light bulbs).
- Some systems require the use of a calibrated blackbody (a tool for checking the calibration of an infrared temperature sensor) during evaluation to make sure measurements are accurate.

#### 6.1.4. Thermal Imaging in COVID-19

In the current COVID-19 pandemic, thermal cameras are receiving increasingly more interest. According to the U.S. Food and Drug Administration (FDA) [93], when used correctly, thermal imaging systems have been shown to accurately measure surface skin temperature without being physically close to the person being evaluated. Thermal imaging systems offer certain benefits in that other methods need a closer proximity or contact to measure temperature (for example, non-contact infrared thermometers or oral thermometers).

The FDA has issued guidance [94] to provide a policy to help expand the availability of telethermographic systems (thermal imaging-based systems) used for body temperature measurements for triage use over the duration of the public health emergency.

Telethermographic systems are capable of determining skin-surface temperature at a reference body site (e.g., oral, tympanic membrane, inner canthus) when coupled with software programs able to detect facial features such as the eyes, nose, and mouth. The resultant value allows estimations of core body temperature. One advantage of using telethermographic systems for initial temperature assessment is the potential use in high throughput areas (e.g., airports, businesses, warehouses, factories) and in settings where other temperature assessment products may be in short supply. As the screening system could potentially run autonomously and without the need for an attendant, time spent waiting for screening would see an overall reduction. The available scientific literature supports the use of telethermographic systems in the context of initial human temperature measurement during such a triage process [95]. Additionally, international standards and scientific literature have described guidelines for using telethermographic systems for initial temperature assessment for triage use and best practices for standardized performance testing of such products [96,97].

Thermal imaging techniques have been effectively trialed for the COVID-19 screening purposes recently. Existing thermal imaging tools coupled with the state-of-the-art machine learning algorithms have shown promising results for elevated temperature screening scenarios. Here, we discuss a few of them.

**Figure 10:**
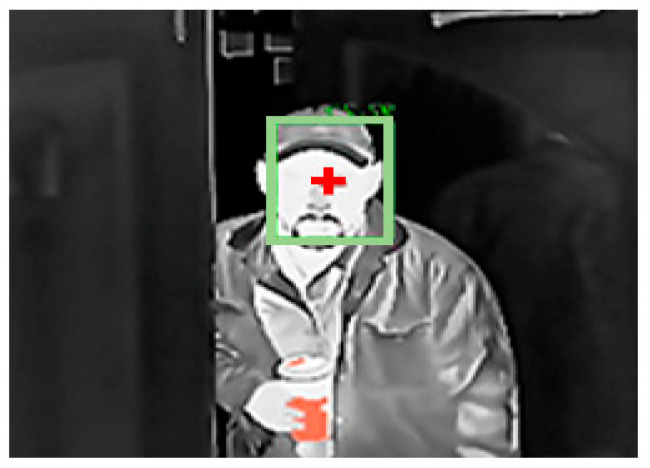
The thermal image presented in [98]. The face area is detected using a face detection algorithm and a point on the forehead (red color “+” mark) is selected for temperature measurement. Image courtesy of een.com.

In a recently released white paper [98], Eagle Eye Networks presented that their tested cameras consistently reported temperatures within +/− 0.7 degrees Fahrenheit of measurements taken with a traditional thermometer. They conducted the experiment for approximately four weeks, testing thermal cameras for elevated temperature screening in some real-world situations as well as lab comparisons. The testing was done with individuals and pairs of subjects (Figure 10). As subjects entered the building, their temperatures were automatically captured by multiple thermal cameras. The thermal camera results were less than the handheld thermometer. The differences ranged from −0.4 to −1.0 degrees Fahrenheit (average difference of −0.7 degrees).

A drone-based networked system was built to combat the COVID-19 pandemic [99]. The thermal imaging system was the most notable feature of their solution. They used this feature to capture the images of people and implement social distancing measurements and for density-based thermal imaging. The drone was tested for COVID-19 operations in the Delhi/NCR, India.

Different organizations and workplaces look for solutions to prevent spreading the virus while making sure their employees and customers feel comfortable. Some real-world examples of using thermal screening for COVID-19 prevention are listed below.

**Figure 11:**
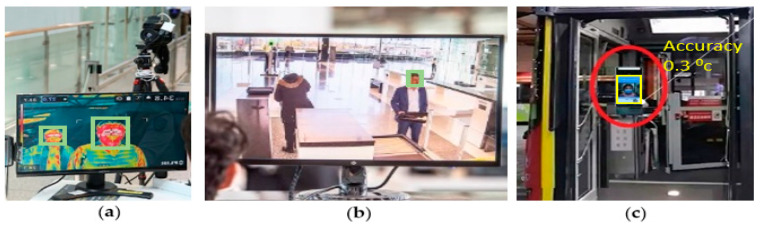
Airports are testing thermal cameras and other technology to screen travelers for COVID-19. (**a**) The thermal screening setup at Incheon International Airport [102], (**b**) a similar setup at Canberra international airport [103], and (**c**) thermal camera setup in public transport [105].

- Amazon Inc., Tyson Foods Inc., and Intel Corp have started to use thermal cameras at their warehouses to speed up screening for feverish workers who could be infected with the coronavirus [100].
- Thermal camera systems have been deployed in several international airports such as Dulles international airport [101], Hong Kong international airport [101], Incheon international airport [102], and Canberra international airport [103] (Figure 11).
- Asian cities have introduced temperature-check kiosks and smart bus shelters to make public transport safer [104,105] (Figure 11).
- Universities have introduced thermal temperature screening stations [106].

Gostic et al. [107] analyzed the effectiveness of different traveler screening programs to limit further global spread of COVID-19. Thermal scanners used for the screening were one of their parameter values. They reported a sensitivity of infrared thermal scanners for fever detection was 70% with 60–90% being the plausible range. Similar studies estimated sensitivity between 60–88% [108,109,110]. However, a handful of studies estimated very low sensitivity (4–30%). In general, sensitivity depended on the device used, body area targeted, and ambient temperature.

### 6.2. Video Camera Imaging Technology

**Figure 12:**
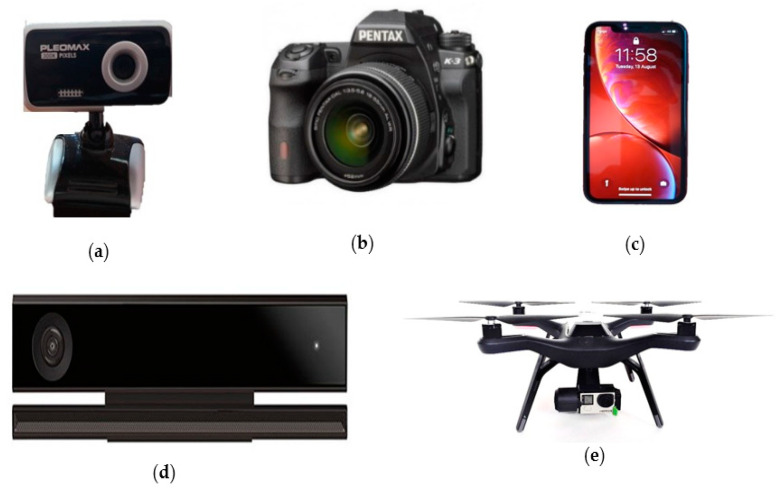
Different sensors used as a video camera. (**a**) Webcam, (**b**) digital camera, (**c**) smart phone, (**d**) Microsoft Kinect, and (**e**) UAV.

Video imaging is a passive and non-contact modality that can be delivered from common sources of video data including hand-held and fixed video cameras, webcams, smartphones, or from sensor platforms such as unmanned aerial vehicles (UAV) and robots, shown in Figure 12. Video analysis of vital signs generally relies on two phenomena. The first phenomenon known as color based methods or imaging photoplethysmography (iPPG) depends on skin color variations caused by cardiorespiratory activity. The second phenomenon, known as motion based methods, relies on cyclic body motion such as the motion of an arterial pulse, head movements, or movements within the thoracic and abdominal regions due to cardiorespiratory activity.

Video cameras have been shown to be able to measure heart rate in controlled clinical settings, in the outdoors, and from multiple people and at long range [32]. Recent work has demonstrated breathing rate in challenging scenarios such as from a drone camera platform [111]. Video cameras have the ability to both diagnose and identify people, which can be useful in many scenarios [112]. Their cost is low, their installed base is large, and the availability of video cameras that can be pressed into service is high.

Video cameras are often integrated with a microphone that might allow detection of coughing [113,114,115]. An important advantage of video cameras is the possibility of using advanced image processing techniques to detect posture and to recognize if a patient is coughing. From longer ranges, individuals in acute distress might be detected by their movements or if they should collapse [111].

A significant advantage of video cameras over all other sensors, including thermal cameras, is their ability to have arbitrary field of view, with the same camera capable of imaging bacteria and the ice caps of Mars with a change of lens [32].

#### 6.2.1. Vital Signs Measured with a Webcam

**Figure 13:**
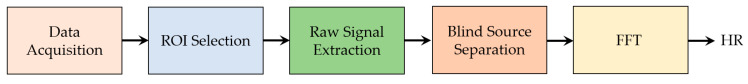
Block diagram of non-contact HR monitoring system proposed in [116].

Webcams have been used as low-cost video cameras to monitor vital signs in literature, as shown in Table 3. Blind source separation (BSS) was a common technique used in different studies to reduce motion artefacts. For example, Pho et al. [116] presented a robust and automatic technique using a webcam for monitoring heart rate based on blind source separation. As shown in Figure 13, in the proposed method, ROI was selected automatically using the Viola–Jones [117] algorithm. The raw signal was obtained using spatial averaging, and independent component analysis (ICA) was applied to get the desired source signal. Finally, FFT was done to attain a frequency spectrum where the frequency with maximum power in the spectrum was considered as the pulse signal. It was possible to monitor three subjects simultaneously using the proposed algorithm. However, only small movements and no illumination variations were considered in the proposed work. The second component obtained by ICA was considered as the desired source signal every time, which might be limiting. Purche et al. [118] and Feng et al. [119] also measured heart rate by means of a webcam based on ICA. Lewandoska et al. [120] introduced another non-contact method to monitor HR via a webcam, based on principle component analysis (PCA). They stated that PCA is better than ICA in terms of computational complexity and time; therefore, PCA can be an alternative technique if only heart rate needs to be measured. Nevertheless, subjects were instructed to be still during the data collection.

Using a webcam, Bousefsaf et al. [121] extracted both instantaneous HR and RR considering normal head movements based on continuous wavelet transform (CWT). Wu et al. presented a time-frequency analysis technique using CWT [122] and a motion resistant spectral peak tracking (MRSPT) technique [123] to monitor heart rate using webcam considering seven situations including participants who were running, driving, and doing fitness training. To extract heart rate from moving subjects using a webcam, Feng et al. [124] proposed a robust monitoring system based on adaptive color variation between green and red channels, followed by an adaptive bandpass filter (ABF). However, the above discussed methods are susceptible to illumination variation and limited to a short distance.

**Figure 14:**
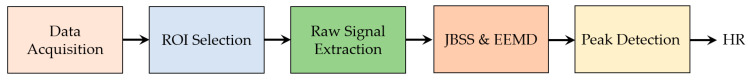
Block diagram of noncontact HR monitoring system proposed in [125].

Signal decomposition is a common technique used to reduce illumination variations in literature. Cheng et al. [125] introduced a robust technique to measure heart rate using a webcam. To reduce illumination variations, the authors combined both joint blind source separation (JBSS) and ensemble empirical mode decomposition (EEMD) and considered background images as well, shown in Figure 14. However, the authors mainly considered controlled illumination variations, which confines the real time applicability of the proposed technique. Another study by Xu et al. [126] tried to minimize the effect of illumination variations by combining partial least squares (PLS) and multivariate empirical mode decomposition (MEMD) to measure heart rate by means of a webcam under varying illumination scenarios. However, only artificial illumination produced by an LED lamp was considered in the proposed method. The above two methods only considered illumination variations, but were susceptible to motion artefacts and again confined to short distances.

#### 6.2.2. Vital Signs Measured with Digital Cameras

**Figure 15:**
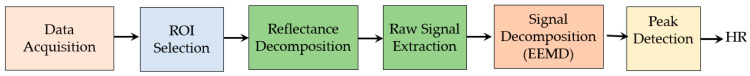
Block diagram of non-contact vital sign monitoring system proposed in [127].

Digital cameras are the most popular means of capturing videos for monitoring vital signs. Chen et al. [127] introduced a robust system to extract heart rate using a digital camera based on reflectance decomposition on the green channel and signal decomposition based on EEMD to reduce the noise due to illumination variations, as shown in Figure 15. However, EEMD can misinterpret periodic illumination variations as physiological signals, particularly when the frequency is close to the normal pulse frequency range, specifically from 0.75 to 4 Hz. This system was further improved in [128] based on a multiple linear regression (MLR) model and Poisson distribution to suppress the effects of ambient light variation. Nevertheless, both systems are not appropriate for real time applications.

Lee et al. [129] proposed a different technique to measure heart rate using multi-order curve fitting (MOCF) to minimize noise artefacts, and recorded video of participants using digital cameras while they were watching television in a dark room. To remove the noise caused by illumination variations, the brightness signal was subtracted from the raw signal. Tarassenko et al. [130] demonstrated a novel technique using auto-regressive (AR) modelling and pole cancellation to suppress the aliased frequency components due to artificial light flicker. However, the above methods only considered illumination variations but affected by motion artefacts.

Using a digital camera, Al-Naji et al. [131] monitored cardiorespiratory signals combining both EEMD and ICA to reduce noise artefacts caused by both illumination variation and motion artefacts.

Arts et al. [132], Cobos-Torres et al. [133], and Gibson et al. [134] also used digital cameras to monitor heart and respiratory rates of infants in the neonatal intensive care unit (NICU).

A charged couple device (CCD) and complementary metal-oxide-semiconductor (CMOS) cameras were used by De Haan et al. [135,136], Wang et al. [137,138,139], and Yu et al. [140] to monitor vital signs. However, most of the above discussed methods have limitations including short measurement range, considering either motion artefacts or illumination variations and a single participant at a time.

#### 6.2.3. Vital Signs Measured with Other Sensors

The built-in camera of a smartphone was used by Kwon et al. [141] to monitor heart rate, and they developed an iPhone-based application, FaceBEAT, for remotely monitoring heart rate. Bernacchia et al. [142] extracted heart rate and respiratory rate using a Kinect device based on ICA. Smilkstein et al. [143] and Gambi et al. [144] also exploited Microsoft Kinect device for measuring heart rate using the Eulerian video magnification technique (EVM).

**Figure 16:**
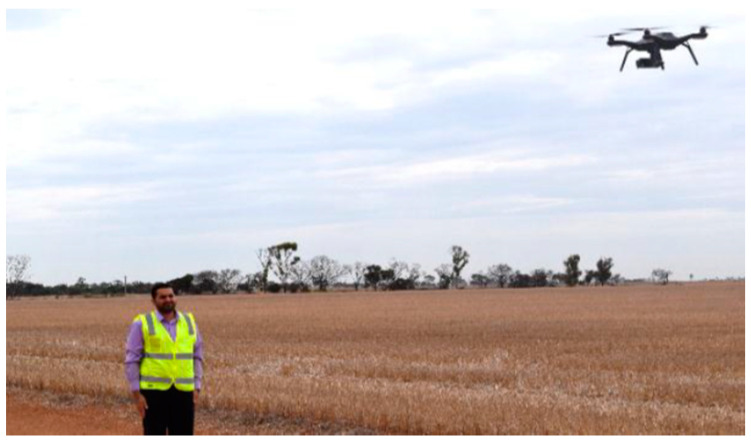
Video recorded by the UAV (3DR solo drone) [145].

**Figure 17:**
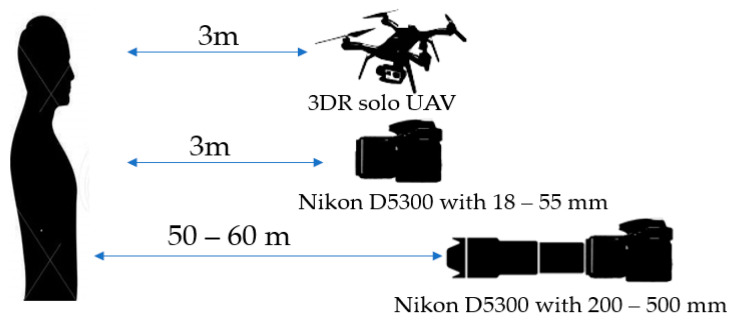
Scenarios for remote optical detection of vital signs [146].

**Figure 18:**
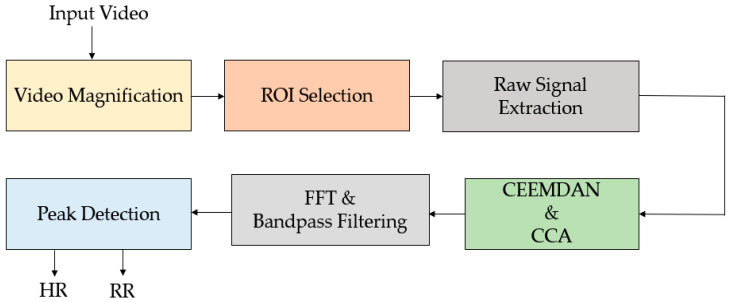
Block diagram of non-contact vital sign monitoring system proposed in [146].

Al-Naji et al. [145] first used a hovering UAV, as shown in Figure 16, to remotely monitor heart and respiratory rate based on improved video magnification technique. The authors used advanced signal processing techniques including signal decomposition using complete EEMD (CEEMD) and BSS using ICA to reduce noise artefacts caused by motion artefacts and illumination variations. A robust non-contact technique was proposed in [146] to calculate cardiorespiratory signal via a digital camera as well as a hovering UAV. Figure 17 shows scenarios for remote optical detection of vital signs. Drone cameras have successfully measured human vital signs, while facing challenges in image stability and vibration. Using stationary cameras with short focal lengths, there has been consideration of extending the capability to multiple people who might be talking and moving. With longer focal lengths, issues of vibration, optical quality, and atmosphere start to dominate. In the proposed method, first, video is magnified using video magnification technique (Figure 18). Then, a ROI was selected, and raw cardiorespiratory signal was extracted. To remove noise artefacts caused by illumination variations and movement of subjects and cameras, the authors used a noise reduction technique integrating both complete ensemble empirical mode decomposition with adaptive noise (CEEMDAN) and canonical correlation analysis (CCA). For spectral analysis, FFT was used, and for filtering, two Butterworth bandpass filters were applied. Finally, the MATLAB built-in function “findpeaks” was used to find the number of peaks to measure heart and respiratory rates. Using the proposed method, it was possible to measure vital signs of six people simultaneously, with a range of measurement of up to 60 m, under both stationary and non-stationary situations. Moreover, a graphical user interface (GUI) was introduced that enabled a user to load video data, select the type of magnification, and execute the proposed algorithm.

### 6.3. Combinations of Different Technologies

To enhance the performance of monitoring vital signs, different technologies can be combined, as shown in Table 4. For example, by combining an RGB camera, a monochrome camera with color filter, and a thermal camera, a study by Gupta et al. [147] proposed an efficient multicamera measuring system to measure HR and HRV. In the proposed method, face segmentation was done using conditional regression forests (CRF) to detect facial landmarks in real-time, and an ROI was selected. The raw signal was calculated based on a spatial average of the pixels within the ROI for each channel, i.e., red, green, blue, magenta, and thermal (RGBMT). To recover the underlying source signals, ICA was used, and a bandpass filter was applied to the selected source signal. Finally, peak detection was performed to calculate HR and HRV. The experimental results showed that the GRT (green, red, and thermal) channel combination gave the most accurate results, demonstrating that the inclusion of more spectral channels could attain more robust and accurate measurements.

Hu et al. [148] presented a dual-mode imaging technique based on visible and long-wave infrared wavelengths to extract breathing rate and pattern remotely using both an RGB and a thermal camera. After performing image registration, to identify a ROI in the RGB image, the cascade object detector based on the Viola–Jones algorithm and the screening technique using biological characteristics were used. Next, to select the corresponding regions in the thermal image, linear coordinate mapping was applied. To select the interest points from ROIs in the visible light gray images, the Shi–Tomasi corner detection method derived from the Harris–Stephens was applied. Cross-spectrum ROI tracking was attained using linear coordinate mapping. After that, the mean pixel intensity was obtained within the ROI of the thermal image. In all of the frames, the raw pixel intensities of ROIs were smoothed by the moving average filter and smoothing method. Finally, breathing rate was extracted from the smoothed signal. By adding RGB images, it was possible to detect and track the face and facial tissue in thermal images more accurately and faster. However, the above systems may not be feasible for measuring vital signs at night.

Another dual camera imaging system was proposed in [149] to measure BR and HR at night using an RGB infrared camera and thermal imager. Image registration was done based on affine transformation, face detection was performed using a pre-trained boosted Cascade classifier or fully convolutional network, and a discriminative regression-based approach was used to select landmarks on the face. IR images were used to select ROI from thermal images using linear coordinate mapping. ROI tracking in thermal images was done by spatio-temporal context learning (STC). A moving average filter was applied to the raw signal and finally, HR and BR were calculated.

Bennett et al. [150] used both a thermal and optical camera to monitor heart rate and blood perfusion based on EVM. After identifying ROI from both thermal and optical videos, the average intensity was computed within the ROI. After that, a Butterworth lowpass filter was used to filter these signals, and then normalized. The experimental results of blood perfusion showed that optical video with EVM was sensitive to skin color and lighting conditions, while thermal video with EVM was not sensitive to these variables. However, in the proposed method, movement of the subject was not considered, and the sample size was small. Although a temperature response caused by blood occlusion was sensed by the thermal camera, it was not in the pulsatile manner of perfusion.

## 7. Hypoxemia Detection Using Video Cameras

Blood oxygen saturation (SpO_2_), alongside heart rate, respiratory rate, and body temperature, is an important vital sign. It is a relative measure of the amount of oxygenated hemoglobin with respect to the total amount of hemoglobin in the blood. It shows whether an individual has an adequate supply of oxygen, hence indicating the condition of the cardiorespiratory system. In detecting hypoxemia, it is important to monitor the level of oxygen saturation continuously.

In recent years, different studies used video cameras to measure blood oxygen saturation remotely either using ambient light or a dedicated light source based on the ratio-of-ratios principle that calculates relative pulsatility at different wavelengths. Some studies used monochrome cameras and others used RGB cameras to capture video of participants.

### 7.1. SpO_2_ Measured with Monochrome Camera

Wieringa et al. [151] first introduced an “SpO_2_ camera” technology using a CMOS camera and LED-ringlight at three wavelengths of 660, 810, and 940 nm. However, they did not present any SpO_2_ results due to the poor signal-to-noise ratio of the photoplethysmographic signals they obtained. Humphreys et al. [152,153] also demonstrated a noncontact pulse oximeter using a CMOS camera and LED arrays at two different wavelengths (760 and 880 nm). Though the authors did an experiment on ten volunteers, it was not possible to measure SpO_2_ accurately due to large noise in the PPG signal caused by the low frame rate and illumination variations.

Another study by Kong et al. [154] first proposed a noncontact scheme to screen blood oxygen saturation using two CCD cameras, individually mounted with a different narrow band pass filter at two different wavelengths, 520 and 660 nm, under ambient light. The experimental results showed good agreement between the proposed method and the reference pulse oximeter. However, the study only covered a small SpO_2_ range (97–99%). Moreover, the proposed method was highly susceptible to motion artifacts. On the other hand, Saho et al. [155] presented a contactless technique for monitoring SpO_2_ using a CMOS camera and an illumination system including two identical LED arrays varying at two wavelengths, 611 and 880 nm. To evaluate the proposed technique, they did an experiment over an SpO_2_ range of 83–98%, and experimental results were consistent with those obtained by a pulse oximeter. However, the proposed method required a dedicated light source, which could irritate the participants. Moreover, the system was constrained by motion artefacts, light scattering, and short range potential.

Verkruysse et al. [156] investigated the feasibility of calibrating noncontact pulse oximetry using two monochrome cameras, individually mounted with spectral bandpass filters at center wavelengths of 675 nm (red) and 842 nm (IR), respectively. They did an experiment on 26 healthy subjects under hypoxic and normoxic situations (83–100%) and slow cooling. However, the proposed method was highly affected by low pulsatile strength and motion artefacts. Another study by Moço et al. [157] also assessed the viability of calibration of contactless SpO_2_ using the ratio of ratios of red and green light. They used three monochrome cameras, respectively, mounted with bandpass filters with green, red, and near-infrared wavelengths of 580, 675, and 840 nm to capture the video of 46 healthy volunteers during hypoxic and normoxic situations (85–100%) and under slow cooling. The authors demonstrated that SpO_2_ could be calibrated with red and green light under controlled settings; however, the accuracy was less than that measured in the typical red-NIR spectrum.

Gastel et al. [158] first introduced a motion-robust contactless monitoring system to measure SpO_2_ based on a new principle using an adaptive PBV method. They used three identical monochrome cameras each equipped with optical filters with a center wavelength of 760, 800, and 840 nm. To validate the method, they did a test on healthy moving volunteers whose SpO_2_ levels varied from 80 to 100%, caused by breath-holding actions in order to induce hypoxemia. The experimental results showed that the new principle outperformed the existing noncontact ratio-of-ratios based approaches. However, the number of participants considered to evaluate the proposed method was very limited.

### 7.2. SpO_2_ Measured with RGB Camera

Instead of using a monochrome camera, a few researchers used RGB cameras to monitor SpO_2_ based on normalized ratio of red and blue channel. For example, Tarassenko et al. [130] introduced a noncontact method to monitor SpO_2_ using a digital video camera under ambient light. Unlike others, they did a large sample size experiment on 46 patients in the Oxford Kidney Unit, whose SpO_2_ varied from 87% to 95%. However, calibration was a big issue for calculating SpO_2_. Using a webcam and ambient light, Bal et al. [159] calculated SpO_2_ based on a skin detector to only identify skin pixels from the selected face region. To evaluate their method, they did a test on six healthy participants and three paediatric intensive care unit (PICU) patients with results showing a strong correlation in comparison with a Masimo oximeter, covering the SpO_2_ range between 93% and 99%.

Another study by Guazzi et al. [160] proposed a novel method to monitor changes in oxygen saturation using an RGB camera and two LED lighting panels in a controlled environment. An automated ROI-selection process was adopted by calculating signal-to-noise ratios for each ROI. To validate the proposed method, an experiment was done on five healthy participants considering an SpO_2_ range of 80–100% for 40 minutes each in a purpose-built chamber. However, the proposed method was affected by factors such as illumination variations, camera spectral responses, and physiological issues like skin type and melanin concentration. A low cost noncontact method was introduced by Rosa et al. [161] using a Raspberry Pi camera and ambient light to monitor SPO_2_ based on the Eulerian video magnification (EVM) method. The EVM was employed to amplify the light absorption variations of red and blue channels. The proposed algorithm showed good accuracy in comparison with those measured with a commercial pulse oximeter. However, the method was constrained to SpO_2_ range 92–99%, a small population, and short distance.

From the above discussion, we have found that most of the methods used a commercial pulse oximeter as a ground truth for validation purposes, which itself has some errors. Moreover, most of the methods were constrained to a small test population, motion artefacts, illumination variations, short distance, and a small range of SpO_2_. It is necessary to be able to monitor SpO_2_ over a broader range (at least 80% to 100%) for real applications, especially for clinical applications. A controlled hypoxemia test could be helpful to drive more variation in the oxygen level of blood.

## 8. Acoustic Detection of Respiratory Infection

Cough detection via acoustic sensing has been a topic of interest for many years. This approach is relatively non-intrusive, and can be completely non-contact, requiring only the presence of an acoustic sensing device within a reasonable proximity (primarily governed by the level of background noise) and a device to facilitate the requisite signal processing. Some implementations have demonstrated use of a contact microphone [162,163,164,165]; however, many modern approaches have adopted the ubiquitous smart-phone for audio capture (and in some instances, also processing) device [166,167,168,169]. A cough event lasts somewhere between 300 [170] and 650 ms [171], and can be described by three distinct phases: the expulsive phase, the intermediate phase, and the voiced phase [172].

It is common to divide the cough detection system into two distinct stages, data pre-processing for feature extraction and machine learning for classification. The primary difference between the approaches are the choice of features and the type of network used for inference. A commonality observed in the data pre-processing stage is the down-sampling of the input signal. Many recording devices capture audio at 44.1 kHz. It appears that this is unnecessarily high for cough detection, with authors reporting success with 22 kHz [171], 16 kHz [173], 11.25 kHz [170], and even down to 400 Hz [168]; each was able to achieve a classification accuracy of greater than 90% through various methods. A down-sampled signal reduces the computational requirements in the subsequent feature extraction process; thus, it is beneficial for embedded applications.

The features extracted for the classifier vary between applications. A recent implementation shown in [170] provides a good example of the diversity in features. The signal was first split into 75 ms windows, with 19 ms overlap (to avoid boundary effects). The justification for this window size was that it allows accurate spectral estimation, while accounting for the non-stationary nature of the signal. Each 75 ms frame was broken down into five frequency bands, ranging from 0 to 5.525 kHz (without overlap). A feature set was extracted from each band in a given frame, giving the spectral centroid, bandwidth, crest factor, flatness, flux, roll-off, peak energy, Renyi entropy, kurtosis, skewness, 90% to 50% energy frequency ratio, relative power, harmonic ratio, Mel-frequency cepstrum coefficients, audio spectrum flatness, normalized audio spectrum envelope, tonal index, chromatic entropy, and sub-band spectral centroid histograms. A total of 117 features were extracted for each window; however, feature selection was applied to remove redundant sources of information, reducing the number of features to 29. A support vector machine (SVM) was used for training and classification, yielding accuracy of around 90% sensitivity, and 80% specificity.

A similar approach can be seen in Matos et al. [162], who used a keyword-spotting approach to detect cough sounds with a hidden Markov model (HMM) classifier. The work in [164] uses spectral, noise, and prosody features for feature extraction and tests their efficacy with three separate classifiers: an artificial neural network (ANN), Gaussian mixture model (GMM), and SVM. It was shown that the GMM performs best for most feature sets. There are other applications, seen in [165,166,168,171,174,175,176,177,178], which use some subset of the above mentioned features (except for [173], which uses gammatone frequency cepstral coefficients), in conjunction with varying machine learning classifiers such as the logistic regression model [178], k nearest neighbour [166], binary tree classifier [168], SVM [178], and convolutional neural network (CNN) [171,177].

It is noted that in most cases, a combination of spectral and cepstral features is extracted from sub-band frames. The cepstrum is typically used for speech detection but also appears to contain a significant amount of information pertaining to the detection of coughs, due to its prevalence in the literature. Interestingly, there are very few applications that make use of the CNN architecture, despite its efficacy in relevant fields such as environmental sound classification [179], heart sound feature extraction [180], snore sound classification [181], and bird sound classification [182]. In such applications, it is common to simply use the mel-spectrogram of the audio signal as the two-dimensional input to the network [179]. It is suggested that various techniques may also be used to augment the dataset, such as time stretching, pitch shifting, dynamic range compression, and the addition of background noise [179]. Such techniques may be transferred to a cough-detection data set, which is inherently sparse due to the nature of the signal [173]. Recurrent neural networks may also be a viable alternative; although being more computationally intensive, they are proven to perform well in speech recognition tasks [183].

It is noted that there are several limiting factors that affect the efficacy of cough detection systems in practice. The signal to noise ratio is often much higher in training data than in practice, due to the nature of the data collection process. It is recommended that training data contains a significant amount of noise, as it is common for other sounds such as laughter, sneezing, talking, and throat clearing to be detected as false positives [164,169]. The specificity of the network to a given person and recording device also pose limitations when applied to new scenarios. The work presented by Barata et al. [171] addressed the issue of device specificity by capturing samples with many different types of microphone. The results indicate a reduced classification accuracy (by approximately 5%) when the classifier is run on recordings from a microphone not used as part of the training process. This reinforces the need for a diverse training set if the developers seek hardware abstraction. Computational resource requirements pose limitations to any embedded real-time application. Testing has not been conducted with CNN architectures; however, it is noted that the standard approach of spectral and cepstral feature extraction and classification can be heavily resource-consuming, costing computational resources and battery life [166]. This is an impediment to embedded mobile applications and must be taken into consideration during the design process. Larson et al. [169] demonstrates a unique approach to this problem. The spectral and cepstral features are extracted from the mobile device, and sent to a cloud-based classifier, thus reducing the amount of processing required by the embedded system.

As a final note, relevant to the current situation, it is possible that further development could help to discriminate between a productive cough and an unproductive cough. An example of such work can be seen in Murata et al. [184], which, if successfully implemented, could assist in discriminating between subjects with COVID-19 and other illnesses.

## 9. Video Based Cough Detection

Many cough detection studies are focused on detecting the cough using the sound the person makes. Sometimes, this is not reliable, for example, in cases where there is no sufficiently strong sound signal to detect. Such scenarios may include people coughing while covering their mouth or being too weak to make a sound while coughing.

Video based cough detection provides an alternative to such problems and can also be used to cross-validate the cough detection of acoustic models. Here, we are reviewing the few notable studies focused on cough detection using visual features.

Cough recognition is widely studied under general action recognition. Some popular action recognition datasets contain coughing action classes. NTU RGB + D dataset [185] has included coughing or sneezing clips in a separate class in their datasets. UWA3D dataset [186] also contains a coughing action class of 71 clips. ALMOND [113] is a dataset created by merging several action recognition datasets. Its coughing/sneezing class contains a total of 1019 clips collected from NTU RGB + D and UWA3D datasets. Thi et al. [114] presented a new dataset targeting sneezing and coughing detection in videos. Their Sneeze–Cough dataset was created to help recognize flu-like behavior symptoms in public areas. The dataset contains 960 video clips collected from 20 human subjects (eight females and 12 males) of 20 to 50 years old.

Compared to acoustic cough detection, a relatively low number of studies have been conducted for video-based cough detection. A cough counting study was performed by Smith et al. [187] by comparing video and audio recordings. They studied eight patients with chronic cough overnight in laboratory conditions. Coughs were recorded simultaneously using a video camera with infrared lighting and digital sound recording. They proposed to use ambulatory digital audio recordings as the gold standard for ambulatory validation of automated cough monitoring devices. Another notable study was performed to recognize flu-like symptoms from videos [114]. The authors developed a new family of kernels that explicitly integrate space–time layout and Bag-of-Words representations. The proposed algorithm was evaluated on the new Sneeze–Cough dataset. Buzzelli et al. [113] demonstrated a relatively similar but more generalized vision-based system for monitoring common actions of elderly people at home. The proposed method consisted of deep learning models for subject localization and action recognition.

**Figure 19:**
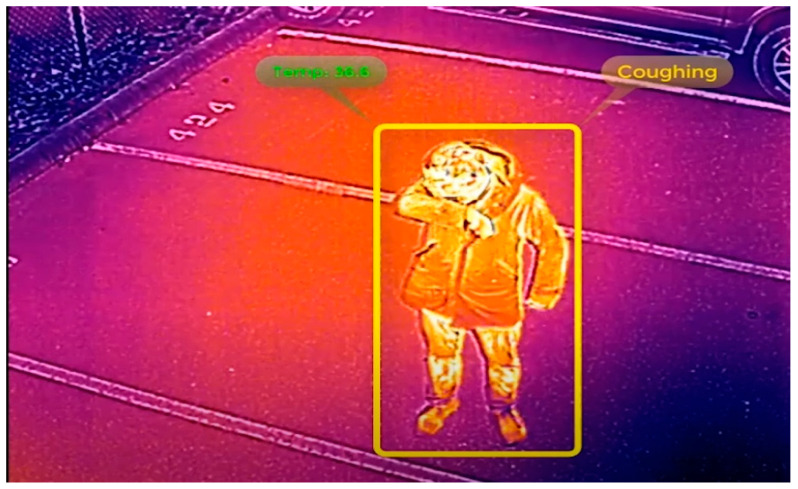
Cough detection using drones. Image courtesy of Draganfly Innovations Inc.

Cough detection using drones has gained the attention of drone manufacturers [188]. Identifying subjects with coughing symptoms from the crowds could be helpful to detect potential patients with respiratory illnesses [189] (Figure 19). High-resolution cameras, faster data transmission links, high performing computers, and action recognition technologies could be effectively used for such solutions.

## 10. Discussion

We have shown that camera imaging techniques using thermal cameras and video cameras, separately or in concert, have been considered for measuring physiological parameters and symptoms of illness. Most of the techniques have been developed in laboratory and clinical environments and could best be described as “non-contact” rather than remote. In general, measurements are under controlled lighting, indoors and at close range, with some notable exceptions.

The challenge is to achieve detection over ranges more associated with the field of noncontact sensing, from long range and moving platforms. In the remote sensing field, achieving mapping or survey objectives is common, yet the research considering non-contact sensing of illness was focused on small areas, static observation, and an approach focused on the individual rather than the ailment. Even the studies with drone measurement of cardiorespiratory parameters were done at quite close range, albeit limited mainly by the characteristics of the modest sensors used.

No concerted effort to develop sensing of this most devastating of situations, a coronavirus, influenza, or any other type of pandemic, has been undertaken. From the literature we find not efforts to undertake such missions, but rather technological indications that there is an underlying feasibility. The domain seems to be wide open to research in noncontact sensing, but the technological hurdles are significant.

We have shown that at short range acoustics might serve to detect coughs and perhaps sneezes. At longer ranges, it should be possible to detect the actions associated with coughing, and by analogy sneezing.

Thermal cameras have the potential to measure all likely symptoms on a single camera, especially temperature, yet they are expensive and currently of comparatively low resolution. Research to date, and the approved usage of such devices, is based on short ranges with thermal calibration and a reliance on detecting anomalous individual temperature, rather than absolute temperature. Range will continue to be limited by the resolution of thermal sensing technology and focusing elements. Thermal imaging techniques are susceptible to slight heat variations and some other complex factors such as head rotation and motion artefacts. Moreover, these techniques are also susceptible to environmental thermal noise caused by the variations of background temperature.

Video cameras, due to their availability, cost, adaptability, and compatibility, are an attractive option, but they will not be able to directly sense temperature and might at best be able to infer it from elevated heart rate or breathing rate of a population compared to statistical norms. The resolution potential of visible light imaging means that they will be useful for long range detection of symptoms that cause gestures, postures, or gaits.

All imaging techniques that use intensity information, thermal imaging, and color based techniques require a clear ROI. These techniques may not always be feasible if a subject is covered by a blanket, face mask, or intubation equipment. Motion-based techniques can be used in such situations, although they may be more limited in range and measurement scenarios, particularly if the sensing platform is in motion.

At this stage, comprehensive studies of COVID-19 noncontact sensing have yet to be done, although there has been a substantial deployment of thermal imaging equipment for screening purposes. It is worth considering some appropriate configurations of COVID-19 sensing for different scenarios, based on the analysis above.

It is likely that in exigent circumstances, a normal visible light webcam can provide some primary indications of COVID-19 symptoms, particularly with a subject at rest, and particularly if they have a history of resting vital signs recorded. Heart rate, SpO_2_, and breathing rate parameters are all measurable and indicative of different aspects of the illness. The microphones on most web cameras also allow for cough detection, another strong indicator of the illness. This solution has the advantage of cost, potentially less than $20 USD when used with an existing computing device. Mobile telephones have both cameras, computing and microphones. Options exist with this solution being universally accessible.

A more comprehensive solution might consist of a LWIR camera with or without a black body calibration device. Frequently, these solutions include a visible light camera to classify objects and parts of the body, particularly the face. This combination of sensors might just be used to measure temperature, but advanced systems might include software to measure vital signs using a combination of thermal and visible image processing. At present, these sorts of systems are expensive, requiring expensive thermal cameras, dedicated computing, and a dedicated platform.

## 11. Conclusions

Noncontact sensor modalities, especially thermal and video cameras, could provide a technique to screen large numbers of people for fever, cough, increased respiratory rate, and heart rate with no risk to operators. Such techniques might provide more sensitive screening methods for detecting COVID-19 than the current method of screening for fever alone. A more useful outcome would be new methods for control and response to contagion in human populations through surveillance and mapping.

Our review has found that the principles of noncontact detection have been demonstrated in indoor or highly controlled environments and mostly at quite short range. The challenge to the noncontact sensing community is to extend these techniques to longer ranges and make them robust enough to be effective on mobile and airborne platforms. The topic is of critical importance and represents a significant research opportunity given the unprecedented harm done to the world’s economy, society, and stability by the pandemic.

## Figures and Tables

**Table 1:** Different studies to extract body temperature using thermal cameras.

Ref	Sensor Used	ROI	Used Technique	Temperature Measured
Bilodeau et al. [41]	7.5–13 μm, LWIR (FLIR ThermoVision A40M)	Face	Particle filter, Kalman filter	-
Aubakir et al. [42]	8–14 μm, LWIR (FLIR Lepton 2.5)	Forehead	V-J method	34.95 °C to 37.00 °C
Sharma et al. [43]	7.5–13 μm, LWIR (FLIR X63900) NIR (CP-PLUS CP-USC-TAL2)	Face	V-J method	29.45 °C to 32.82 °C
Lin et al. [44]	8–14 μm, LWIR (FLIR Lepton 2.5) 8–14 μm, LWIR (KeySight Keysight U5855A)	Forehead	Deep- learning	27 °C to 37.5 °C
Sumriddetchkajorn et al. [45]	7.5–13 μm, LWIR (FLIR ThermoVision A40M)	Face	Image filtering, particle analysis	35 °C to 40 °C
Silawan et al. [46]	8–14 μm, LWIR (Optris PI450)	Forehead, mouth, cheek	Multiple data comparison	36.0 °C to 39.5 °C
Thomas et al. [47]	7.5–14 μm, LWIR (Fluke TiS65)	Face	Linear regression	34 °C to 41 °C

Note: NIR = Near infrared, LWIR = Long wavelength infrared.

**Table 2:** Different studies to monitor vital signs using thermal cameras.

Ref	Sensor Used	Vital Signs	ROI	Used Technique	Result
Murthy et al. [50]	3–5 μm, MWIR (hardware unspecified)	BR	Nose	Advanced statistical algorithm	Accuracy = 98.5%
Fei et al. [51,52]	3–5 μm, MWIR (FLIR model unspecified) 3–5 μm, MWIR (Indigo Systems model unspecified)	BR	Nose	Optical bandpass filter	-
Sun et al. [53]	MWIR (Indigo Systems model unspecified)	HR	Forehead, neck and wrist	FFT	PCC = 0.994
Garbey et al. [54]	MWIR Indigo camera (Indigo Systems model unspecified)	HR	Forehead, neck and wrist	FFT	CAND = 88.52%
Chekmenev et al. [55]	LWIR (FLIR model unspecified)	HR and BR	Face and neck	CWT	-
Fei et al. [56]	3–5 μm, MWIR (FLIR SC6000)	BR	Nose	CWT	CAND = 98.27%
Shakhih et al. [57]	7–14 μm, LWIR (Infrared Camera Incorporation 7640 P-series)	TI and TE	Nose	Mean pixel intensity	PCC = 0.796 (TI), 0.961 (TE)
Pereira et al. [58]	7.5–14 μm, LWIR (VarioCAM R HD head 820S/30)	BR	Nose	Particle filter framework and temporal filtering	MAE = 0.33, 0.55 and 0.96 breaths/min.
Pereira et al. [59]	2–5.5 μm, MWIR (InfraTec 9300)	HR and BR	Head and nose	Particle filter framework, temporal filtering and PCA	RMSE = 3 bpm (HR), RMSE = 0.7 breaths/min.
Pereira et al. [60]	7.5–14 μm, LWIR (VarioCAM R HD head 820S/30)	BR	Nose, mouth, shoulders	Particle filter framework and signal fusion	RMSE = 0.24,0. 89 breaths/min.
Abbas et al. [61]	1–14 μm, LWIR (VarioCAM HR head)	BR	Nose	CWT	-
Pereira et al. [62]	7.5–14 μm, LWIR (VarioCAM R HD head 820S/30)	BR	Nose	Particle filter framework and temporal filtering	Relative error = 3.42%
Pereira et al. [63]	7.5–14 μm, LWIR (VarioCAM R HD head 820S/30)	BR	-	Black-box	RMSE = 4.15 ± 1.44 breaths/min.

Note: MWIR = Mid wavelength infrared, LWIR = Long wavelength infrared, MAE = Mean absolute error, RMSE = Root mean square error, CAND = Complement of the absolute normalized difference.

**Table 3:** Different studies to monitor vital signs using video camera technology.

Ref	Sensor Used	Vital Signs	ROI	Used Technique	Results
Pho et al. [116]	Webcam	HR	Face	ICA	PCC = 0.95, RMSE = 4.63 bpm
Purche et al. [118]	Webcam	HR	Forehead, nose and mouth	ICA	
Feng et al. [119]	Webcam	HR	Forehead	ICA	PCC = 0.99
Lewandoska et al. [120]	Webcam	HR	Face and forehead	PCA	
Bousefsaf et al. [121]	Webcam	HR	Face	CWT	
Wu et al. [122]	Webcam	HR	Face	CWT	SNR (dB) = −3.01
Wu et al. [123]	Webcam	HR	Cheeks	MRSPT	RMSE = 6.44 bpm
Feng et al. [124]	Webcam	HR	Cheeks	GRD	PCC = 1
Cheng et al. [125]	Webcam	HR	Face	JBSS + EEMD	PCC = 0.91
Xu et al. [126]	Webcam	HR	Face	PLS + MEMD	PCC = 0.81
Chen et al. [127]	Digital camera	HR	Brow area	EEMD	PCC = 0.91
Lin et al. [128]	Digital camera	HR	Brow area	EEMD + MLR	PCC = 0.96
Lee et al. [129]	Digital Camera	HR	Cheek	MOCF	RMSE = 1.8 bpm
Tarassenko et al. [130]	Digital camera	HR, RR, SpO_2_	Forehead and cheek	AR modelling and pole cancellation	MAE = 3 bpm
Al-Naji et al. [131]	Digital camera	HR and RR	Face, palm, wrist, arm, neck, leg, forehead, head and chest	EEMD + ICA	PCC = 0.96, RMSE = 3.52
Arts et al. [132]	Digital camera	HR	Face and Cheek	JFTD	-
Cobos-Torres et al. [133]	Digital camera	HR	Abdominal area	Stack FIFO	PCC = 0.94
Gibson et al. [134]	Digital camera	HR and RR	Face and chest	EVM	Mean bias = 4.5 bpm
De Haan et al. [135]	CCD	HR	Face	CHROM	PCC = 1, RMSE = 0.5
De Haan et al. [136]	CCD	HR	Face	PBV	PCC = 0.99, RMSE = 0.64
Wang et al. [137]	CCD	HR	Face and forehead	2SR	PCC = 0.94
Wang et al. [138]	CCD	HR	Face	POS	SNR (dB) = 5.16
Wang et al. [139]	CCD	HR	Face	Sub-band decomposition	SNR (dB) = 4.77
Yu et al. [140]	CMOS	HR and RR	Palm and face	SCICA	PCC = 0.9
Kwon et al. [141]	Smartphone	HR	Face	ICA	MAE = 1.47 bpm
Bernacchia et al. [142]	Microsoft Kinect	HR and RR	Neck, thorax and abdominal area	ICA	PCC = 0.91
Smilkstein et al. [143]	Microsoft Kinect	HR	Face	EVM	-
Gambi et al. [144]	Microsoft Kinect	HR	Forehead, cheeks, neck,	EVM	RMSE = 2.2 bpm
Al-Naji et al. [145]	UAV	HR and RR	Face	CEEMD + ICA	PCC = 0.99, RMSE= 0.7 bpm
Al-Naji et al. [146]	Digital camera, UAV	HR and RR	Face and Forehead	CEEMDAN + CCA	PCC = 0.99, RMSE= 0.89 bpm

**Table 4:** Different studies to monitor vital signs using combined technology.

Ref	Sensor Used	Vital Signs	ROI	Used Technique	Result
Gupta et al. [147]	RGB, monochrome and thermal camera	HR and HRV	Cheeks and forehead	ICA	Error = 4.62%
Hu et al. [148]	RGB and thermal camera	BR	nose and mouth	Viola–Jones algorithm together with the screening technique	LCC = 0.971
Hu et al. [149]	RGB infrared and thermal camera	HR and BR	Mouth and nose regions	Moving average filter	LCC =0.831 (BR), LCC = 0.933 (HR)
Bennett et al. [150]	Thermal and digital camera	HR and blood perfusion	Face and arm	EVM	-

Note: LCC = Linear correlation coefficient.

## Data Availability

Not applicable.
